# Effect of Baduanjin Qigong on Sleep Quality and Hyperarousal State in Adults With Chronic Insomnia: Protocol for a Randomized Controlled Trial

**DOI:** 10.2196/53501

**Published:** 2023-12-12

**Authors:** Chaoqun Xie, Fangfang Xie, Jianwen Ma, Hongyu Yue, Yanli You, Fei Yao

**Affiliations:** 1 School of Acupuncture-Moxibustion and Tuina, Shanghai University of Traditional Chinese Medicine Shanghai China; 2 Department of Acupuncture-Moxibustion Tuina and Rehabilitation Center, Shanghai Municipal Hospital of Traditional Chinese Medicine Shanghai China; 3 Department of Traditional Chinese Medicine, Changhai Hospital, Naval Medical University Shanghai China

**Keywords:** Baduanjin qigong, chronic insomnia, functional magnetic resonance imaging, hyperarousal, randomized controlled trial

## Abstract

**Background:**

Chronic insomnia (CI) is a mind-body disease that is commonly defined as a state of having disturbed daytime activities due to poor nighttime sleep quality. Baduanjin qigong (BDJQG) is widely used for CI in China. However, there is little scientific evidence to evaluate its effects on the hyperarousal state, which is closely associated with improved sleep quality.

**Objective:**

The objective of the trial is to assess the therapeutic effects of BDJQG on sleep quality in patients with CI.

**Methods:**

A randomized controlled trial will be conducted on 86 patients, who will be divided into a BDJQG group and a cognitive behavioral therapy for insomnia group at a ratio of 1:1. Interventions in both groups will be given to the participants 7 times a week for 8 weeks, and the participants will be followed up for 4 weeks. The primary outcome is the change in the Pittsburgh Sleep Quality Index from baseline to week 8. The secondary outcomes are the changes in the Hyperarousal Scale, Insomnia Severity Index, Fatigue Scale-14, wrist actigraphy, salivary cortisol level, and functional magnetic resonance imaging from baseline to week 8. All main analyses will be carried out on the basis of the intention-to-treat principle.

**Results:**

This study was funded from January 2023. As of the submission of the manuscript, there were 86 participants. Data collection began in April 2023 and will end in January 2024. Data analysis is expected to begin in January 2024, with the publication of results expected in February 2024.

**Conclusions:**

This study will present data concerning the clinical effects of BDJQG on CI. The results will help to demonstrate whether BDJQG is an effective therapy for improving sleep quality in association with a decreased hyperarousal level as a possible underlying mechanism. This study will provide much-needed knowledge for complementary and alternative therapy for patients with CI.

**Trial Registration:**

China Clinical Registration Agency ChiCTR2300069241; https://chictr.org.cn/bin/project/ChiCTR2300069241

**International Registered Report Identifier (IRRID):**

PRR1-10.2196/53501

## Introduction

Chronic insomnia (CI) is the most common clinical sleep disorder. It is characterized by persistent challenges in initiating or maintaining sleep; suboptimal sleep quality; and impairments in daytime functioning, including fatigue, attention deficit, and emotional instability [1]. The prevalence of CI in the general population varies between 9% and 12%, with transient insomnia symptoms reported by 22% to 35% of individuals [2]. The widely recognized classification framework for sleep disorders, the *International Classification of Sleep Disorders, Third Edition*, stipulates specific criteria for diagnosing CI. Specifically, the diagnosis of CI necessitates the presence of insomnia symptoms for a minimum duration of 3 months, with no fewer than 3 associated symptoms, which may encompass manifestations such as fatigue, irritability, and compromised work, school, or social performance [3]. While CI is classified as a sleep disorder, its pathophysiological underpinnings suggest a prevailing state of hyperarousal during both sleep and wakefulness phases [4]. Evidence of the hyperarousal phenomenon in individuals with insomnia encompasses elevated levels of cortisol and adrenocorticotropic hormone during the initial stages of sleep and discernible thalamic responses determined through functional magnetic resonance imaging (fMRI) [5].

Several pharmaceutical agents, including benzodiazepines, benzodiazepine agonists, and certain antidepressants, have demonstrated efficacy in the short-term management of insomnia [6]. However, these pharmacological interventions carry inherent risks related to the sedative properties. The risks encompass an elevated propensity for falls; cognitive confusion; and the potential for the development of tolerance, dependence, and withdrawal effects when used for extended periods [7]. Cognitive behavioral therapy for insomnia (CBTI), regarded as the first-line treatment for insomnia, has proven its effectiveness in addressing CI in adults. It consistently ameliorates clinical symptoms associated with insomnia, producing clinically meaningful effects [8]. Nevertheless, the accessibility of CBTI remains challenging for patients, primarily due to the limited availability of specialized practitioners, which hinders its ability to adequately meet clinical demands [9]. Several complementary and alternative medicine modalities, including traditional Chinese herbal medicine [10], acupuncture [11], and qigong, have exhibited efficacy in the treatment and prevention of insomnia, effectively mitigating symptoms such as fatigue and depression.

Qigong is a component of traditional Chinese medicine characterized by its integrated approach, involving breath control, physical movement, and meditation techniques. Notably, extant research has furnished evidence supporting the beneficial effect of qigong on various facets of health among individuals with insomnia, including the enhancement of sleep quality [12,13], alleviation of symptoms related to anxiety and depression [14,15], and modification of sleep-wake patterns as corroborated by actigraphy assessments [16].

The nomenclature “Baduanjin qigong” (BDJQG) was first documented in *Yi Jian Zhi* by Hong Mai during the Northern Song Dynasty, indicating that this mind-body practice has a history spanning over 800 years [17]. This modality is renowned for its holistic approach, characterized by facilitating overall coordination, unblocking meridians, harmonizing the mind and body, and assisting the body in achieving a relative equilibrium between the yin and yang elements. The safety and efficacy of BDJQG have garnered empirical support through numerous clinical trials, rendering it a widely embraced therapeutic approach for diverse chronic ailments [18-20]. For example, an illustrative meta-analysis has indicated that BDJQG, particularly when integrated with mindfulness techniques, is effective in alleviating musculoskeletal pain and enhancing the overall sleep quality in individuals with chronic conditions [21]. Furthermore, BDJQG comprises 8 distinct movements that are easy to learn. If its efficacy is proven, its wider use could be promoted.

Previous investigations into insomnia treatment have primarily centered on enhancing sleep quality but have often overlooked the assessment of daytime functioning. In contrast, our previous research findings suggested that qigong exhibits efficacy both in ameliorating sleep quality and in enhancing daytime functioning [22].

Remarkably, assessments of the hyperarousal state have been conspicuously scarce in previous trials. In this study, we intend to comprehensively evaluate hyperarousal from a multidimensional perspective, encompassing cognitive functions (evaluated through the Hyperarousal Scale [HAS]), cortical activities (scrutinized through fMRI), and hypothalamic-pituitary-adrenocortical (HPA) axis functioning. Salivary cortisol level (SCL), known to correlate with various sleep parameters [23], will be measured, given that hyperarousal in insomnia is associated with heightened cortisol secretion. Previous empirical evidence has directly indicated that BDJQG may induce changes such as increased melatonin levels and decreased cortisol levels, which are indicative of reduced wakefulness-related factors [24]. These physiological markers will be meticulously monitored as potential indicators of BDJQG’s effect on the HPA axis.

Building upon the insights gleaned from previous research and considering the plausible role of BDJQG in attenuating hyperarousal, we have formulated a randomized, open-label, parallel-controlled trial to systematically investigate the therapeutic efficacy of BDJQG concerning both sleep quality and hyperarousal state in individuals with CI.

## Methods

### Aims and Study Design

The primary objectives of this open-label, parallel, noninferiority randomized controlled trial (RCT) will be 2-fold:

1. To ascertain whether BDJQG can yield improvements in the primary outcome, which is sleep quality, and the secondary outcomes, encompassing an array of measures spanning various domains, including hyperarousal state, severity of insomnia, and levels of fatigue. These improvements will be evaluated in comparison to CBTI.
2. To explore the potential efficacy of BDJQG as a therapeutic intervention for CI and its potential association with a reduction in hyperarousal levels, which may serve as an underlying mechanistic pathway. This exploration will be facilitated through correlation analyses.

### Study Setting

Participants will be recruited from the Shanghai Municipal Hospital of Traditional Chinese Medicine. Eligibility for inclusion will be determined by 2 orthopedic surgeons based on predefined inclusion and exclusion criteria. Participants who are determined to be eligible and provide written informed consent will be randomly allocated to either the BDJQG group or the CBTI group in a 1:1 ratio. The BDJQG group will undergo BDJQG treatment, while the CBTI group will receive cognitive behavioral therapy for insomnia. Both groups will undergo an 8-week-long treatment program, consisting of 1 supervised session per week at Shanghai University of Traditional Chinese Medicine and the remaining 6 days of the week completed at home. The study will span a total of 13 weeks, comprising a 1-week-long screening period, an 8-week-long treatment phase, and a subsequent 4-week-long follow-up period.

**Figure 1:**
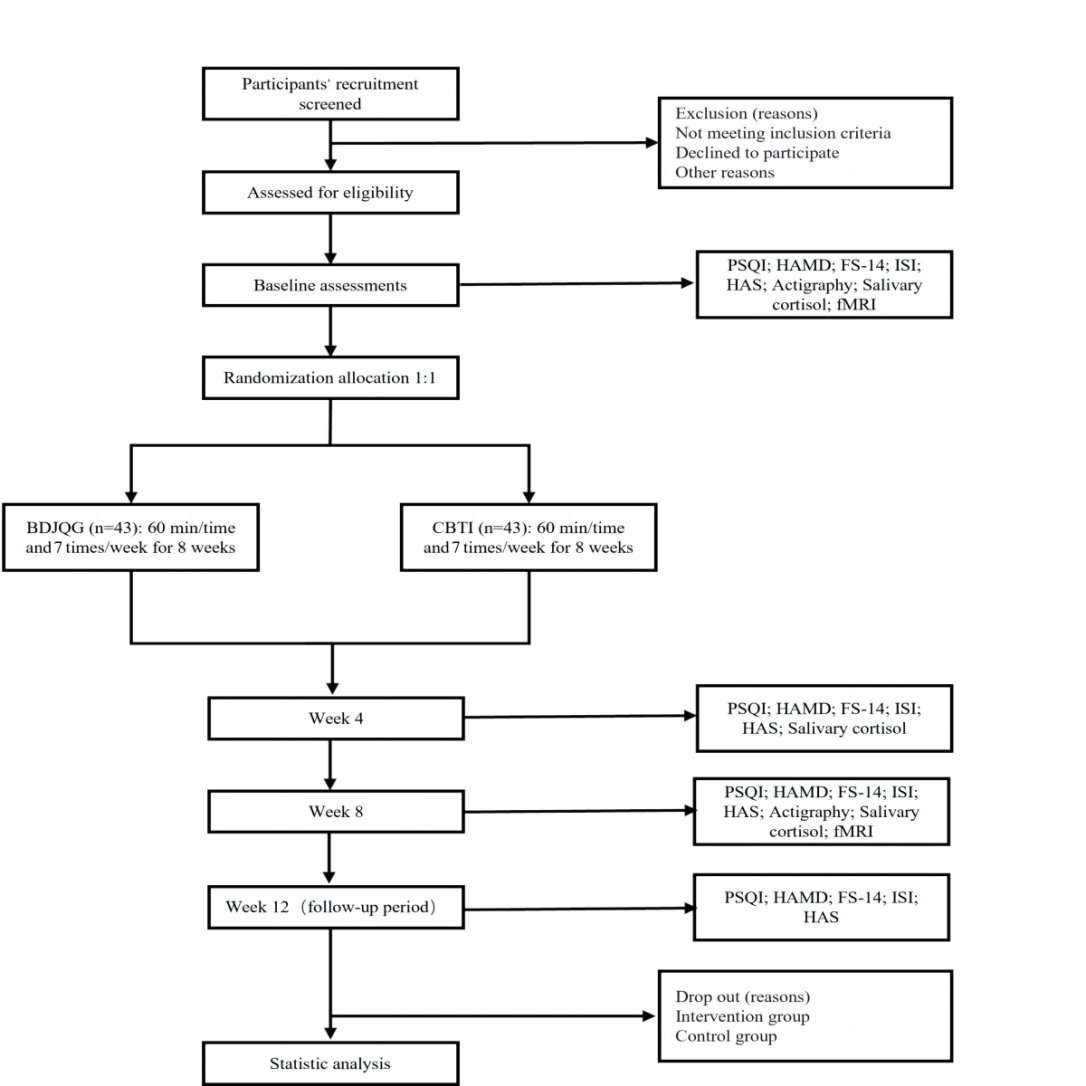
Flowchart of the study design. BDJQG: Baduanjin qigong; CBTI: Cognitive behavioral therapy for insomnia; fMRI: functional magnetic resonance imaging; FS-14: Fatigue Scale-14; HAMD: Hamilton Depression Scale; HAS: Hyperarousal Scale; ISI: Insomnia Severity Index; PSQI: Pittsburgh Sleep Quality Index.

Assessments using various scales will be conducted at baseline, the 4-week intervention mark, the 8-week intervention mark, and the 4-week follow-up. SCL will be measured at baseline, the 4-week intervention mark, and the 8-week intervention mark. Actigraphy and fMRI assessments will be administered at baseline and the 8-week intervention mark. Figure 1 gives a visual representation of the research process, and Table 1 shows an overview of the study’s time points.

**Table 1:** Chart of the trial’s time points.

Research process	Baseline (week 0)	Intervention period (weeks 1-8)	Intervention period (week 4)	Intervention period (week 8)	Follow-up (week 12)
Inclusion criteria	✓				
Exclusion criteria	✓				
Informed consent	✓				
Randomization and allocation	✓				
Intervention		✓			
PSQI^a^	✓		✓	✓	✓
HAMD^b^	✓		✓	✓	✓
ISI^c^	✓		✓	✓	✓
HAS^d^	✓		✓	✓	✓
FS-14^e^	✓		✓	✓	✓
Actigraphy	✓			✓	
Salivary cortisol	✓		✓	✓	
fMRI^f^	✓			✓	
Adverse events		✓		✓	✓
Working practice record		✓		✓	✓

^a^PSQI: Pittsburgh Sleep Quality Index.

^b^HAMD: Hamilton Depression Scale.

^c^ISI: Insomnia Severity Index.

^d^HAS: Hyperarousal Scale.

^e^FS-14: Fatigue Scale-14.

^f^fMRI: functional magnetic resonance imaging.

### Participants’ Eligibility Criteria

Textbox 1 shows the inclusion, exclusion, and withdrawal criteria.

**Inclusion, exclusion, and withdrawal criteria.**

**Inclusion criteria**

Participants meeting the following criteria will be included:

- Chronic insomnia based on established diagnostic criteria (*International Classification of Sleep Disorders, Third Edition*), including a report of sleep initiation or maintenance problems, adequate opportunity and circumstances to sleep, daytime consequences, and duration of at least 3 months, with a frequency of at least 3 times per week
- Insomnia Severity Index score between 8 and 21
- Age between 20 and 55 years and male or female sex
- Hamilton Depression Scale score ≤14
- No treatments for insomnia, including medication and acupuncture, received during the past month
- Right-handedness
- Understanding the process of this study and agreeing to participate and sign the consent form

**Exclusion criteria**

Participants meeting any of the following criteria will be excluded:

- Severe pain or systemic diseases (endocrine system diseases, motor system diseases, autoimmune diseases, infectious diseases, and metabolic diseases such as diabetes mellitus) that affect sleep
- History of metal, head surgery, and implantation of any metal or other contraindications for functional magnetic resonance imaging
- Insufficient language skills and mobility to perform Baduanjin qigong
- Major depressive disorder, anxiety disorder, panic disorder, or other mental disorders, and alcohol or caffeine addiction
- Pregnancy, breastfeeding, or planning to become pregnant

**Withdrawal criteria**

Participants meeting the following criteria will be excluded after the start of the trial:

- Occurrence of an adverse event related to the research
- Participant’s personal request

### Interventions

The two arms of the trial are (1) the intervention arm (BDJQG) and (2) the control arm (CBTI).

#### Intervention Arm (BDJQG Group)

BDJQG consists of 8 forms, which are shown in Multimedia Appendix 1. A qigong teacher from Shanghai University of Traditional Chinese Medicine, who has been involved in qigong education for at least 5 years, will be in charge of the concentrated supervision of the exercise and correction of the exercise posture for 1 hour every Sunday throughout the intervention period. The teacher will first lead a 5-minute stretching and relaxation exercise session to be done before the qigong exercise; and they will then introduce and demonstrate each movement, explain precautions, and answer the participants’ questions for 5 minutes. Then, the teacher will give individual guidance to each participant by correcting their movements. This will last for 30 minutes. Finally, all participants will be told to practice qigong together for 20 minutes. The participants will be asked to practice at home for 30 minutes on the remaining 6 days of the week, to practice on WeChat (Tencent) cluster video at 6 PM daily, and to practice under video supervision. Private video surveillance will be carried out if this is deemed appropriate by an individual participant. A “practice log” will be distributed, to be filled in after each exercise. The entire treatment will last for 8 weeks. The BDJQG intervention will be in line with the descriptions provided in the study by Xie et al [25].

#### Control Arm (CBTI Group)

The CBTI protocols used in this study are based on the comprehensive guide titled *Cognitive Behavioral Treatment of Insomnia: A Session-by-Session Guide* [26]. The participants assigned to the CBTI group will undergo an 8-week-long cognitive behavioral therapy program for insomnia, guided by an experienced psychotherapist, with each session spanning 1 hour and occurring weekly. Additionally, the participants will be provided with a “practice log” to document their activities and progress after each video session.

### Follow-Up Period

An 8-week-long follow-up will be conducted subsequent to the conclusion of the trial, during which the participants will resume their regular lifestyles. However, it is imperative that all of the participants maintain a record of their daily exercise or study activities. To facilitate this, WeChat will be used as a platform for the participants to periodically capture and share photos, providing the researchers with a comprehensive account of their daily exercise or study routines. Upon the culmination of the follow-up period, a reassessment of several metrics, including the Pittsburgh Sleep Quality Index (PSQI), Insomnia Severity Index (ISI), HAS, and Fatigue Scale-14 (FS-14), will be conducted. The objective of this follow-up assessment is to evaluate the enduring or long-term effects of BDJQG on individuals with insomnia, as elucidated in a study conducted by Guan et al [27].

### Quality Control

The specific movements and the corresponding intensity levels used in the BDJQG regimen will be meticulously tailored to individual prescriptions, with variations designed to minimize disparities. Preceding the commencement of the experimental phase, each participant will undergo a structured training program spanning 3 days, administered by a traditional Chinese medicine practitioner with a minimum of 5 years of clinical experience. This training regimen is intended to impart standardized BDJQG movements to ensure uniformity and precision among the participants.

### Outcomes

#### Primary Outcome (PSQI)

The primary outcome is the change in PSQI from baseline to week 8. The PSQI is a comprehensive self-report instrument comprising 19 items aimed at evaluating sleep quality and disturbances experienced within a 1-month time frame. These items collectively contribute to the generation of 7 distinct “component” scores, encompassing assessments of subjective sleep quality, sleep latency, sleep duration, habitual sleep efficiency, sleep disturbances, use of sleeping medication, and daytime dysfunction. The sum of the scores derived from these 7 components yields a singular global score. The total global score spans a range from 0 to 21, with a lower global score indicative of a higher quality of sleep [28].

#### Secondary Outcomes

The secondary outcomes include the changes in ISI, HAS, FS-14, actigraphy, SCL, and fMRI from baseline to week 8.

##### ISI Instrument

The ISI is a self-report instrument encompassing 7 items intended to assess various aspects of insomnia, including the challenges associated with falling asleep and maintaining sleep, overall sleep satisfaction, daytime functioning, impaired attention attributable to sleep disturbances, the degree of distress caused by insomnia-related symptoms, and the level of preoccupation with insomnia-related concerns. Each of these aspects is evaluated on a 5-point scale. The cumulative ISI score ranges from 0 to 28 points and is categorized as follows: 0-7=absence of insomnia; 8-14=mild insomnia; 15-21=moderate insomnia; and 22-28=severe insomnia [29].

##### HAS Instrument

The HAS comprises 26 self-report items designed to gauge a range of cognitive and behavioral factors associated with heightened information processing, propensity for introspection, tendencies to engage in deep emotional reflection, and heightened responsiveness to unforeseen stimuli, all of which are postulated to be indicative of cortical arousal [30]. Elevated scores on this scale are suggestive of an increased state of arousal. Consequently, the HAS serves as a valuable tool for assessing the effect of interventions on cognitive and somatic hyperarousal levels [31].

##### FS-14 Instrument

Fatigue is a prominent consequence of CI, as consistently documented in previous research studies [32,33]. The FS-14 is a self-report assessment tool comprising 14 items designed to capture both physical and mental aspects of fatigue. On this scale, a higher score is indicative of a more pronounced and debilitating level of fatigue.

##### Actigraphy

The ActiGraph GT9X Link (Actigraphy LLC) will be worn on the participants’ wrists to record sleep quality by noting sleep awakenings, total sleep time, sleep onset, sleep latency, and efficiency of sleep. Subsequent analysis of sleep conditions and sleep quality will be executed using the ActiLife6 software (version 6.8.1; Actigraphy LLC) [34]. The participants will be instructed to wear the device continuously for 3 consecutive days, both before and after the 8-week-long intervention period.

##### SCL Measurement

SCL will be quantified using the enzyme-linked immunosorbent assay (ELISA) methodology. Sampling will be conducted at 4 distinct time points during the day, namely upon awakening [35], 30 minutes after awakening [36], before sleep (30 minutes before bedtime) [37], and immediately before bedtime [38] (with the timing adjusted to fall within the range of 9 PM to 1 AM, contingent on each participant’s self-selected bedtime). This collection process will be carried out during 3 distinct cycles, namely at baseline, 4 weeks, and 8 weeks.

Saliva samples will be gathered using a Salivette collection system (hygienic saliva collection; Sarstedt) [36] and stored in a –20 °C freezer until they undergo analysis using the General Cortisol ELISA Kit (RK00639; ABclonal) [39]. The participants will be provided with specific instructions not to engage in eating, drinking, toothbrushing, or strenuous physical activity within the 30-minute interval preceding each saliva sampling session.

##### fMRI Examination Procedure

fMRI data will be acquired from all of the participants using a 3.0-T Trio Siemens System (SIEMENS AG) at the Yueyang Hospital of Integrated Traditional Chinese and Western Medicine affiliated with the Shanghai University of Traditional Chinese Medicine, Shanghai, China. A total of 30 participants will be scanned under the following conditions: repetition time=1900 ms; effective echo times=2.93 ms; sagittal slices=188; thickness/skip=1.2/0.6 mm; field of view=256 × 256 mm^2^; matrix=240 × 256 mm^2^; voxel size=1.0 × 1.0 × 1.0; phase encoding direction=A >> P; and flip angle=90°. The participants will be requested to rest for 10 minutes with their eyes closed, and they will be requested not to think about anything before the scan. They will be asked not to move the head during data acquisition. We will acquire 242 3D image volumes with the following parameters: repetition time=2000 ms; effective echo times=30 ms; section thickness=1 mm; sagittal slices=32; field of view=256 × 256 mm^2^; matrix=64 × 64 mm^2^; and flip angle=90°. Both groups of participants will be examined before and after treatment.

### Sample Size

The following 2 hypotheses are related to the differences between the 2 groups:

- Hypothesis 1: μ_1_= μ_2_
- Hypothesis 2: μ_1_≠ μ_2_,

where μ_1_ is the PSQI mean score of the 8-week treatment in the BDJQG group, and μ_2_ is the PSQI mean score of the 8-week treatment in the CBTI group.

The required sample size has been calculated using an estimation formula based on the difference between 2 sample means and SDs (PSQI, which is assessed using composite points of sleep quality). Therefore, in a study with this sample size, the difference between the BDJQG group and the CBTI group will be considered, and the conservative comparison method by Bonferroni will be adopted. In this study, it is expected that the BDJQG group will be 1.78 points better than the CBTI group in the PSQI scale mean score. The final calculated difference and SD of the 2 groups of the PSQI mean scores were calculated [40]. On the basis of 0.9 power to detect a significant difference (α=.025; 1-sided), 34 participants will be required for each group.

The following formula was used to calculate the sample size in this trial:



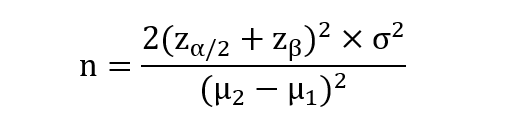



Given that an attrition rate of ~20% is anticipated, our target enrollment will be 43. Therefore, a total of 86 participants should be recruited for this RCT.

### Recruitment

To attain the requisite participant enrollment necessary to achieve our predetermined target sample size, recruitment will be conducted within the Shanghai area. These endeavors will encompass the dissemination of recruitment materials through posters, promotion through WeChat public accounts, engagement with individuals attending outpatient clinics, and outreach to those using medical examination centers.

### Randomization and Blinding

Following a postbaseline assessment, the eligible participants will be randomly allocated to the BDJQG group and the CBTI group, adhering to a 1:1 equal proportion allocation. A randomization list will be meticulously generated by a skilled statistician using a computer program (Strategic Applications Software, version 9.1.3; SAS Institute Inc). The generated random sequences will be sequentially numbered by the statistician, who serves as the architect of the randomization sequence. Subsequently, the sequences will be placed within opaque envelopes by a designated project manager, uninvolved in the recruitment process. These sealed envelopes will ultimately be delivered to the research team.

Before implementing the random assignment procedure, the research team will diligently record the particulars of each participant at the clinical center. These records will encompass the participant’s information, such as name, date of birth, participant and center codes, and the inclusion date. Following a meticulous review of the participant’s information by the research team to ascertain eligibility, a sequence will be randomly drawn from the envelope. The selected sequence will be applied to label the participant’s data, designating the participant’s assignment to one of the study groups. This information will then be conveyed to the respective participants.

It is noteworthy that, due to the specific nature of the intervention approach, this study is an open trial, with only the statistical analysis experts being kept blinded, as delineated in the research conducted by Cheng et al [41].

### Adverse Events and Assessment of Safety

Adverse events (AEs) encompass any untoward and nonsignificant occurrences, namely abnormal experimental findings, symptoms, or illnesses that transpire during the course of the study, irrespective of their causal relation to the intervention. It is important to note that numerous authors have attested to the relative safety of qigong as an approach to addressing various health conditions, including hypertension, with no records of severe AEs. In the event of any unexpected AEs, categorized as functional abnormalities brought about by the intervention, such as symptoms like headache, dizziness, vertigo, head distension, tinnitus, chest tightness, aggravated shortness of breath, heart palpitations, muscular soreness or pain, profuse cold perspiration, irritability, neurasthenia, hallucinations, paranoia, or psychological stress—regardless of their treatment association—prompt notification will be made to the nearest affiliated Shuguang Hospital physician. The physician will then conduct an assessment and initiate appropriate medical intervention.

In instances where serious AEs are encountered, the investigator will promptly report the incident to the primary investigator and the ethics committee to collectively determine whether the affected participant should be withdrawn from the study and the associated treatment. The investigator will earnestly strive to avert and manage any potential harm that may arise during the course of the study. If, following the assessment by the expert committee, the AE is determined to be linked to the qigong treatment, the research team will assume responsibility for covering the cost of treatment and providing appropriate financial compensation for any damage arising from the trial. This comprehensive approach to describing AEs and ensuring the assessment of safety closely adheres to the framework delineated in the study conducted by Guan et al [27].

### Data Management and Monitoring

Upon recruitment, the participants’ identities will be replaced with numerical codes to safeguard their privacy. Data collection will be conducted at baseline, week 4, and week 8. This process will entail the involvement of 2 proficient data specialists who will be responsible for transcribing the paper-based records into a unified database while meticulously verifying the accuracy of the entered data. Ultimately, both the paper records and encrypted electronic files will be securely stored and redundantly backed up by the research leader. These records will be retained in storage for a period of 3 years following the conclusion of the study.

The oversight of the study will be carried out under the purview of the ethics committee of the Shanghai Municipal Hospital of Traditional Chinese Medicine. Importantly, the members of this committee have been carefully vetted to ensure they possess no conflicts of interest pertaining to this study. The principal investigators will retain unfettered access to all study results and will have the final authority to decide on the termination of the study. Furthermore, they will extend the privilege to project team members to disseminate the trial’s findings through the publication of research papers.

### Statistical Analysis

The collected data will meticulously be entered into Microsoft Excel (Microsoft Corporation), and subsequent statistical analysis will be conducted using SPSS software (version 18.0; SPSS Inc). Continuous variables will be summarized using the mean value and SD, with the median and IQR used for variables exhibiting nonnormal distributions. Categorical variables will be presented as frequencies or percentages.

For within-group comparisons, paired 2-tailed *t* tests will be used. Between-group differences will be analyzed through 2-sample, independent-sample *t* tests, with effect sizes computed using Cohen *d* statistics. Furthermore, demographic characteristics, baseline measurements, and changes in measurements from baseline to study completion between the 2 groups will be analyzed using 2-sample, independent-sample *t* tests. In cases where baseline measurements exhibit disparities, analysis of covariance will be implemented to account for these variations when comparing the 2 groups.

The clinical overall efficacy evaluation will be based on a nonparametric test for 2 independent samples. In instances of limited participant numbers within a specific subject or in the presence of missing data, the Mann-Whitney *U* test will be used to assess differences between the groups. A statistical significance level of *P*<.05 will be used, signifying statistical significance when this threshold is met.

### Ethical Considerations

This study will adhere to the principles outlined in the Declaration of Helsinki, ensuring that no additional harm or risks are imposed on the participants. The study has been meticulously designed in compliance with the Standardized Protocol Items, encompassing the recommendations for interventional trials (SPIRIT [Standard Protocol Items: Recommendations for Interventional Trials]) checklist, and has received approval from the ethics committee of Shanghai Municipal Hospital of Traditional Chinese Medicine (2023SHL-KY-17-01). Furthermore, the study has been duly registered in the Chinese Clinical Trials Registry in 2023 (ChiCTR2300069241). The information presented in this report is derived from protocol version 1.0. Before the commencement of the trial, the participants will undergo a comprehensive orientation session regarding the study protocol and their associated responsibilities. This briefing will encompass a detailed explanation of the required physical examinations and relevant precautions. Of paramount importance, the participants will be unequivocally apprised that their engagement in the study is entirely voluntary, and they retain the prerogative to decline participation or withdraw at any juncture without any impact on their medical care or other associated benefits. Notably, if a participant opts to withdraw from the study, any previously collected data will remain intact and be incorporated into the final analysis. To initiate any study-related interventions, explicit written informed consent will be diligently obtained from each participant. A designated research assistant will be entrusted with the responsibility of securing informed consent from all participants. Separate informed consent forms have been developed for the collection of magnetic resonance imaging and saliva samples. Furthermore, all participants will be asked for their consent for the potential publication of identifying information or images within an internet-based open publication, in accordance with established ethical guidelines. The study data will be anonymous or deidentified. Upon the conclusion of the study, the patients will be extended an invitation to continue their 4-week follow-up period at the Shanghai University of Traditional Chinese Medicine, located in Shanghai, China. The 8-week BDJQG program is an enduring initiative, and the participants will maintain their eligibility to engage in this program. Should they desire to do so, the participants can opt to attend BDJQG or CBTI classes free of charge following the culmination of their participation in the study. As an additional incentive to bolster follow-up participation, the participants will be furnished with a large gift pack that will include specialized topical pain patches, a wellness pot, and ¥300 (US $41.98) at the conclusion of the follow-up evaluation. This provision is aimed at enhancing their enthusiasm for continued participation in the follow-up phase.

## Results

This study was funded from January 2023. As of the submission of the manuscript, there were 86 participants. Data collection began in April 2023 and will end in January 2024. Data analysis is expected to begin in January 2024, with the publication of results expected in February 2024.

## Discussion

### Overview

The impact of insomnia extends beyond individual challenges, significantly affecting both personal and societal domains, encompassing physical and mental health, social progress, and economic facets. Regrettably, despite its considerable debilitating effects, effective treatments for insomnia have remained elusive. Moreover, there is limited application of evidence-based interventions aimed at alleviating symptoms and enhancing patients’ overall functioning. Therefore, the quest for a cost-effective and efficacious insomnia remedy is of paramount importance. Numerous studies have underscored the effectiveness of qigong and related therapies in ameliorating insomnia, although these investigations have primarily centered on efficacy and largely overlooked the underlying mechanisms. Furthermore, there is a scarcity of rigorous studies to affirm the clinical effects of BDJQG exercises on insomnia-induced hyperarousal.

The primary objective of this trial is to probe the efficacy of BDJQG in addressing sleep quality and hyperarousal state associated with CI. The trial’s efficacy indicators will encompass both subjective and objective aspects, such as sleep duration and quality as evaluated by the PSQI and wrist actigraphy, daytime functioning as assessed by the FS-14, physiological changes linked to the HPA axis determined through cortisol level measurements, and cortical activities monitored through fMRI. This study aims to establish a connection between the effect of BDJQG on CI and its potential impact on hyperarousal state, measured across multiple dimensions. A diagram to illustrate the mechanism of change before and after the intervention is shown in Multimedia Appendix 2. This novel approach marks the first endeavor in a clinical trial of CI to explore both the clinical effects and the plausible underlying mechanisms associated with BDJQG.

fMRI serves as a vital tool to assess changes in cortical hyperarousal, a phenomenon observed in individuals with insomnia. The thalamus is the switch that controls wakefulness [42]. Patients with insomnia disorder demonstrate decreased thalamic connectivity with the left amygdala, parahippocampal gyrus, putamen, pallidum, and hippocampus across wakefulness [43]. Previous studies have shown that qigong can affect the brain response [22,44-46]. This trial strives to bridge the existing knowledge gap by combining fMRI, which investigates arousal responses spatially within the brain, with wrist actigraphy, enabling the temporal monitoring of arousal status. This combined approach will enhance our ability to comprehensively elucidate the shifts in arousal experienced by individuals with insomnia.

The strengths of this protocol are manifold. First, it represents the inaugural RCT delving into the effects of qigong, specifically BDJQG, on hyperarousal among individuals with insomnia. The findings emerging from this trial will enrich the existing body of research on qigong for the management of insomnia. Second, BDJQG’s accessibility and versatility, enabling indoor or outdoor practice by individuals or groups without necessitating specialized sports facilities or equipment, make it an appealing intervention. Third, BDJQG’s dual benefits of physical and mental conditioning render it suitable for individuals across diverse age groups.

Nonetheless, this study exhibits certain limitations. First, the challenge of achieving blinding in nonpharmaceutical trials, wherein participants are aware of the treatment they receive, could potentially introduce bias into the results. However, rigorous measures have been implemented to ensure that laboratory technicians, data managers, and statisticians remain detached from the recruitment and participant processing phases. Furthermore, recent meta-epidemiological research has indicated no substantial differences in estimated treatment effects between trials with and without patient blinding. Second, the recruitment criteria limit participants to those aged between 20 and 55 years without severe CI, possibly limiting the generalizability of the results to other age groups. Third, as this is a single-center study, it will not recruit participants from diverse regions. Nevertheless, recruitment strategies will encompass community outreach and internet-based channels to diversify the participant pool and minimize regional bias. Therefore, we are prepared to follow up with a multicenter study to provide more adequate evidence.

### Conclusion

This study is poised to provide valuable data regarding the treatment of CI through BDJQG. Its outcomes will help elucidate whether BDJQG is an efficacious approach for enhancing sleep quality and explore its potential mechanistic associations. We will scrutinize the hypothesis that BDJQG’s effect on insomnia may be linked to its capacity to mitigate hyperarousal states. The findings of this trial hold the potential to guide more informed and selective use of BDJQG as an insomnia treatment modality in the future.

## Appendix

Multimedia Appendix 1 The detailed movements of the Baduanjin qigong.

Multimedia Appendix 2 Chart of mechanisms of change before and after the intervention.

## Abbreviations

AE: adverse event
BDJQG: Baduanjin qigong
CBTI: cognitive behavioral therapy for insomnia
CI: chronic insomnia
ELISA: enzyme-linked immunosorbent assay
fMRI: functional magnetic resonance imaging
FS-14: Fatigue Scale-14
HAS: Hyperarousal Scale
HPA: hypothalamic-pituitary-adrenocortical
ISI: Insomnia Severity Index
PSQI: Pittsburgh Sleep Quality Index
RCT: randomized controlled trial
SCL: salivary cortisol level
SPIRIT: Standard Protocol Items: Recommendations for Interventional Trials

## Data Availability

The data sets analyzed during this study are available from the corresponding author on reasonable request.
