# Supplementary material for: Effect of Baduanjin Qigong on Sleep Quality and Hyperarousal State in Adults With Chronic Insomnia: Protocol for a Randomized Controlled Trial
Source: JMIR Res Protoc. 2023 Dec 12;12:e53501. doi: 10.2196/53501 (PMC10751632; doi:10.2196/53501)
Supplement: Multimedia Appendix 1 [file resprot_v12i1e53501_app1.pdf]

Additional file 1.The detailed movements of the BDJQG. (Figure1-8)

Form1: Propping up the Sky

Raise the hands overhead in front of your body with palms facing upward. Turn the palms when they pass by your face. Look upward and lift your heels while propping up. Return to the starting pose by lowering the arms sideways with heels to the ground. Inhale while lifting hands and exhale while lowering them.

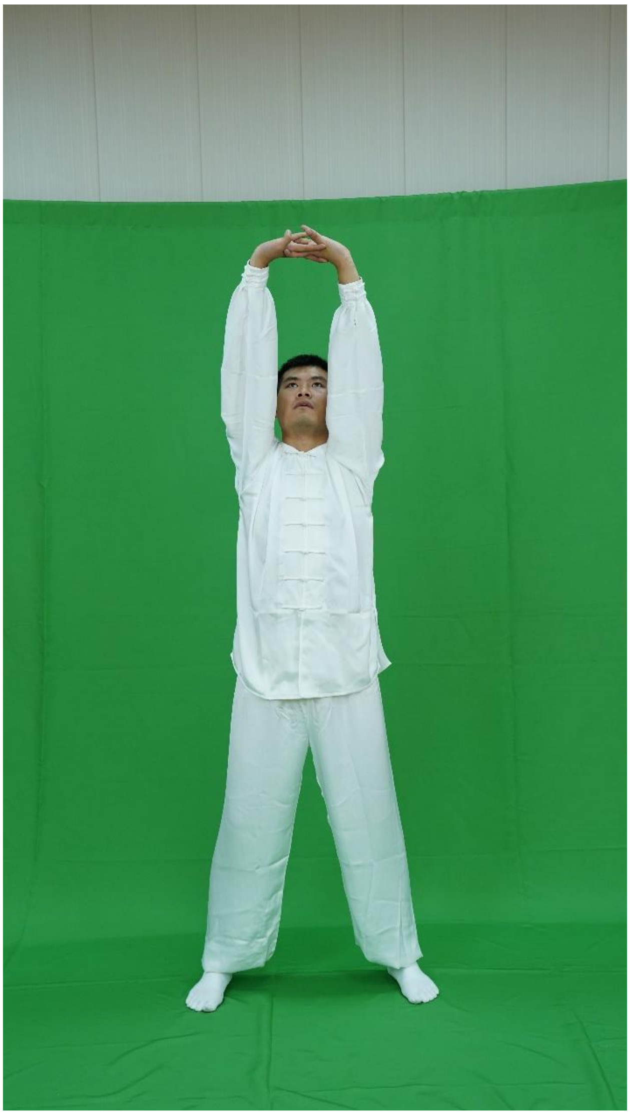

Figure1. Form1: Propping up the Sky

Form2: Drawing the Bow

Take a big side step with feet at two-shoulders' width. Lift your hands, forearms crossed. Stretch out the left arm with the index and the middle fingers erect and other fingers bent. Bend the right arm while making a fist and draw the elbow to the right side. Bend your knees. Repeat this movement to the other side. Inhale when drawing the bow and exhale when return to the starting pose.

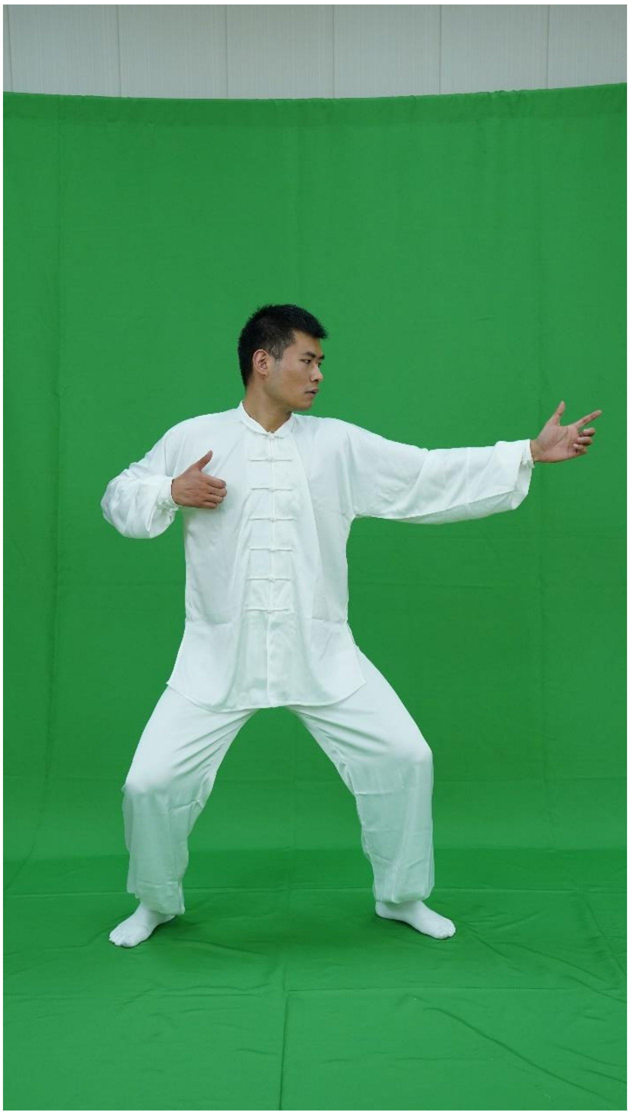

Figure2. Form2: Drawing the Bow

Form3: Raising One Hand

Start with a small step sideways, standing straight in a relaxed way. Lift your hands to the stomach with palms facing upward. Continue the movement by propping the left hand up and pressing the right hand down. Return to the starting pose by lowering the left hand in front of your body. Reverse the action by lifting the right hand. Inhale while lifting the arm and exhale while lowering the arm. Remember to keep the arms vertical, left hand pointing to the right and right hand pointing ahead while stretching the arms.

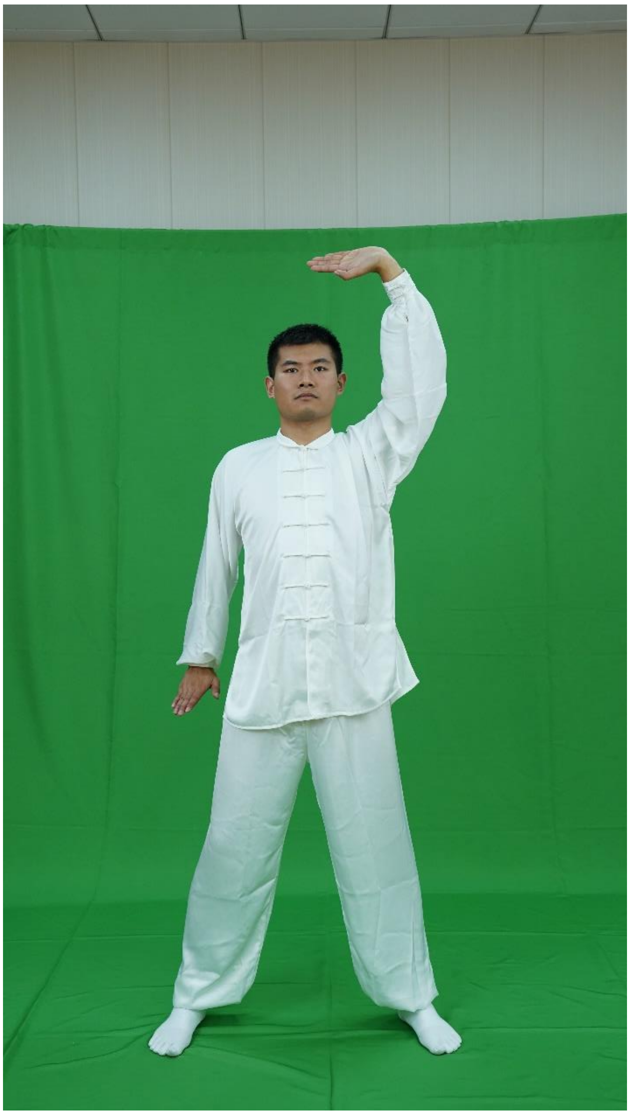

Figure3. Form3: Raising One Hand

Form4: Looking over the Shoulders

Start with a small step sideways. Look ahead with body relaxed. Lift your left arm around the front of your face and hug the back of your head. Lift the right hand to touch your back at the same time. Then clench the right ear with the index and middle fingers of your left hand. Turn the waist to the right and look at the left heel. Return to the starting pose, lowering the left hand around your neck. Reverse this movement. Inhale while lifting your arms and turning your waist. Exhale while returning to the starting pose. Keep the body up straight while looking back.

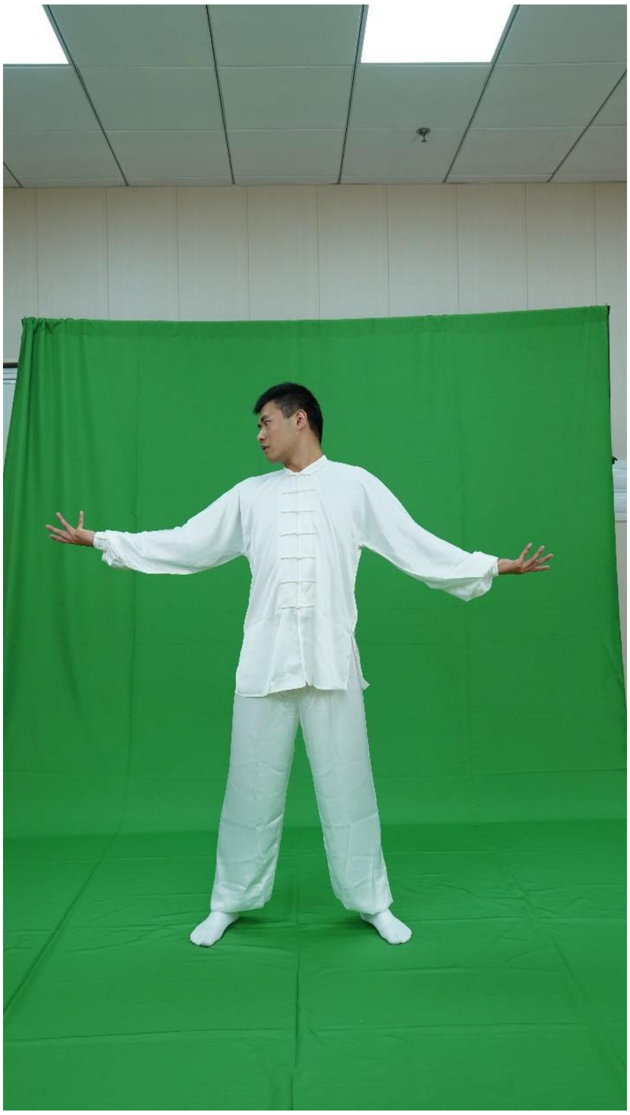

Figure4.Form4: Looking over the Shoulders

From5: Clenching Fists and Looking Forward with Eyes Wide Open

Take a big step sideways and bend down. Let your arms hanging naturally. The first movement is raising and lowering the two shoulders in turn to swivel the vertebrae of the spine. The second movement is swinging the two hands back and forth while swaying the head and hips right and left in opposite ways. The spine weaves at the same time. Breathe naturally.

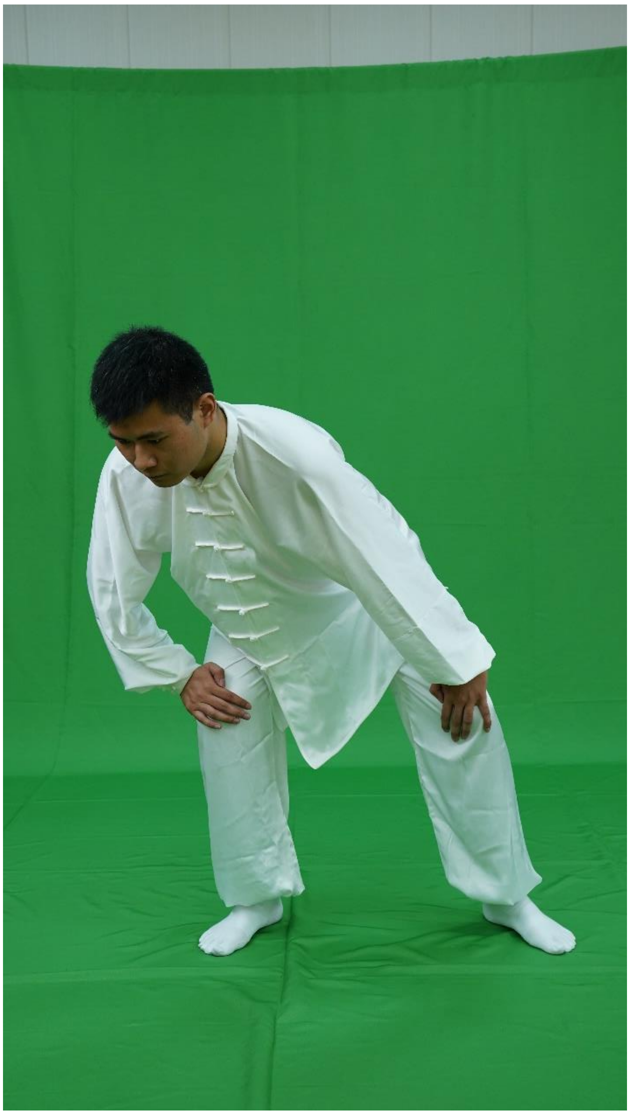

Figure5.From5: Clenching Fists and Looking Forward with Eyes Wide Open

Form6: Pulling the Toes

Stand at ease. Lift your arms overhead and lean backward. Bend forward and try to grasp your toes with your hands. Pull your toes and keep your legs straight. Inhale while leaning backward and exhale while bending down. If you are not limber enough to reach your toes, just stretch the arms toward ground as far as you can.

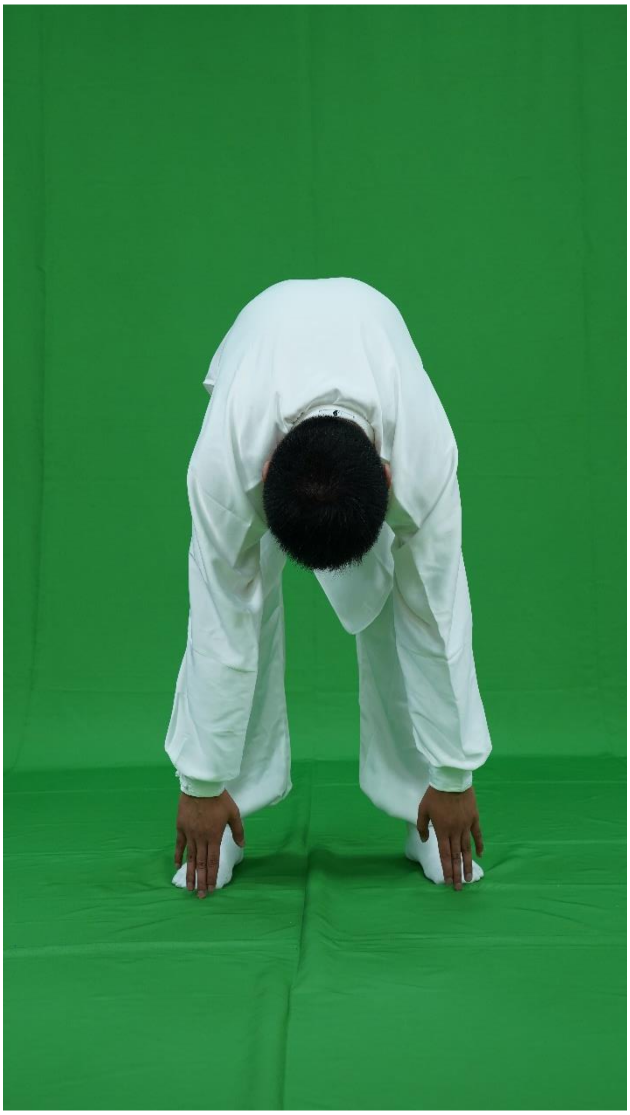

Figure6.Form6: Pulling the Toes

Form7: Swaying Head and Buttocks

Take a big step sideways, feet parallel and at two-shoulders' width apart. Drop the torso with fists beside the waist. Punch a fist slowly forward using internal force. Turn the hand and then withdraw the punched out fist. Punch the two fists in turn. Inhale when withdrawing the fist and exhale when punching. Teeth are clenched tightly and eyes opened wide as if you were angry. Toes should grip the ground firmly. Do not protrude the buttocks and pull in the lower back.

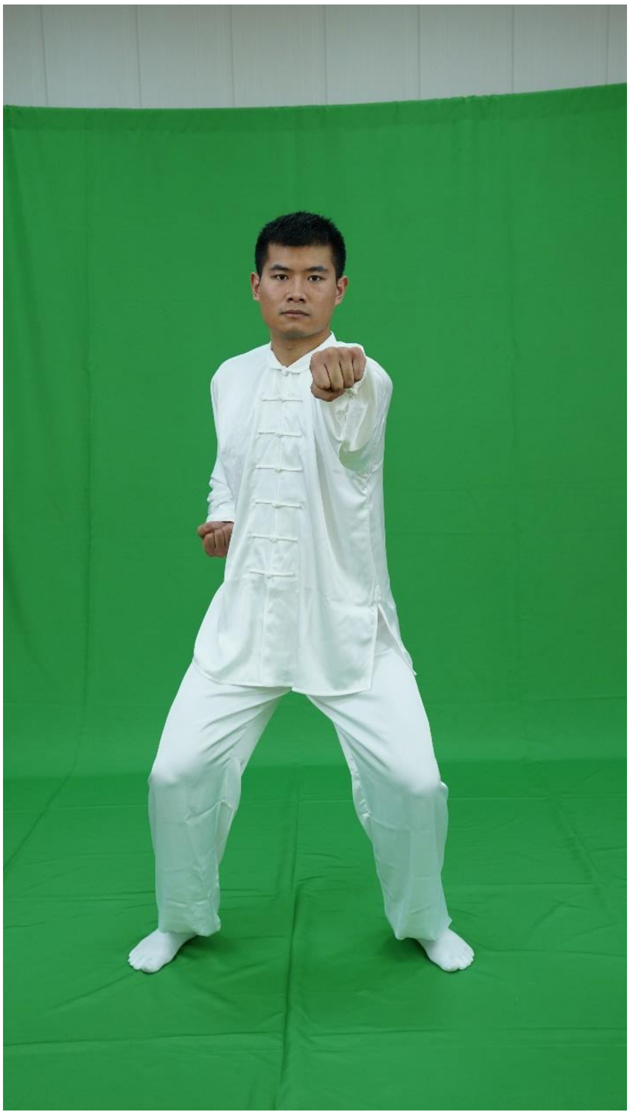

Figure7.Form7: Swaying Head and Buttocks

Form8: Jolting

Stand at ease. Lift your heels and raise your body. Lower the heels suddenly to give your body a little shock. Inhale while lifting the heels and exhale while lowering them. For those who are not very strong, lower the heels gently instead of suddenly. The head should be kept erect to prevent too much shock to the cervical vertebrae.

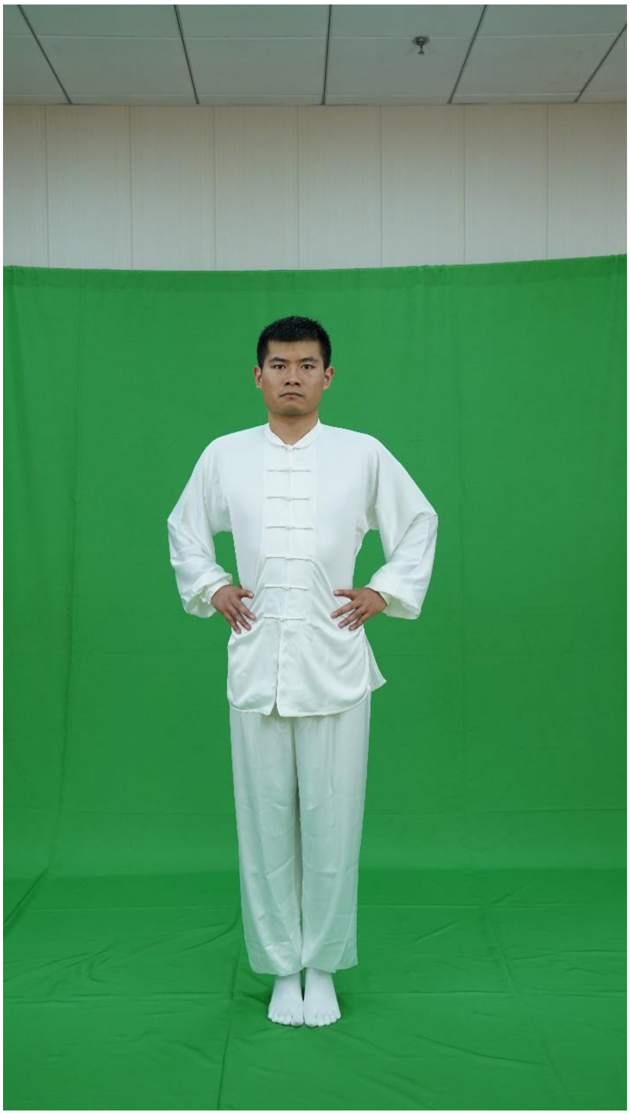

Figure8. Form8: Jolting
