# Supplementary figures and images for: Effect of Baduanjin Qigong on Sleep Quality and Hyperarousal State in Adults With Chronic Insomnia: Protocol for a Randomized Controlled Trial
Source: JMIR Res Protoc. 2023 Dec 12;12:e53501. doi: 10.2196/53501 (PMC10751632; doi:10.2196/53501)

Additional file2.Chart of mechanisms of change before and after the intervention

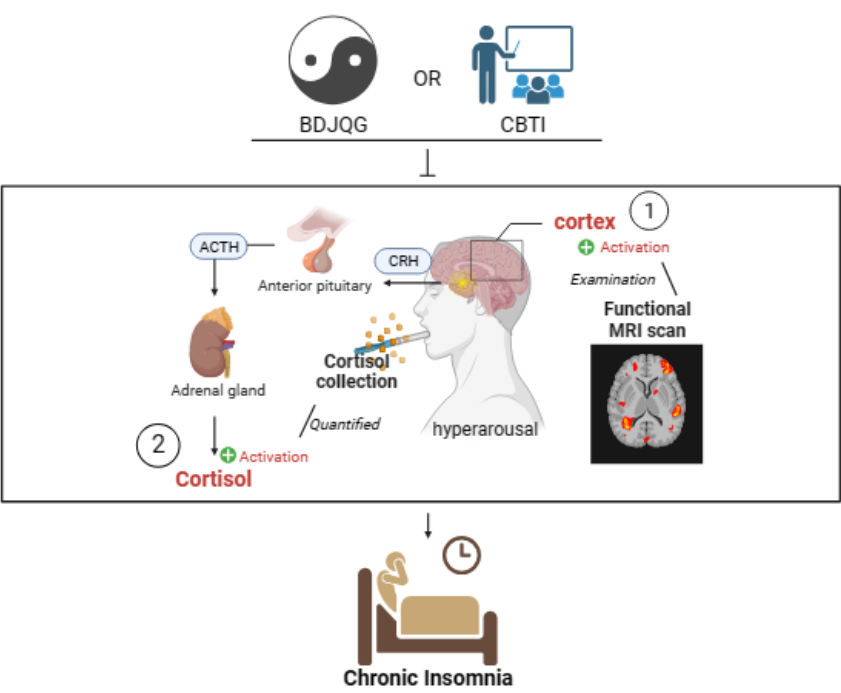

Supplement: Multimedia Appendix 2 [file resprot_v12i1e53501_app2.pdf]
